# 

**Published:** 1891-06

**Authors:** 


CONTENTS.

VOL. 38. No. 6.

| | PAGE. |
|---|---|
| Hints for Hot Weather | 121 |
| Perspiring Feet | 122 |
| Variety in Food | 123 |
| Weak Knees | 124 |
| Putting Away Winter Clothing | 125 |
| Poisoned Air | 126 |
| Mole on Face | 127 |
| The Corset Once More | 127 |
| What the Danish Women Learn | 129 |
| Precious Stones of the Month | 131 |
| A Chinese Custom | 132 |
| The Brain | 132 |
| The Infant Brain | 133 |
| Medicinal Properties of Food | 134 |
| Ringworms and Eczema | 135 |
| Treatment of Slight Wounds | 136 |
| The Ascending Spirit | 137 |
| Blindness in New-Born Infants | 138 |
| Mosquitoes | 139 |
| Faith Cure and Cancer | 139 |
| Cure for Burns | 139 |
| How to be Beautiful | 140 |
| Stuttering Cured | 140 |
| Open Competitive Examination of M. D's | 144 |
| Miscellaneous | 140 |
| Literary | 142 |
| Almost Time | 144 |

INDEX TO ADVERTISEMENTS OF HALF A PAGE AND OVER.

| | PAGE. |
|---|---|
| Horsford's Acid Phosphate | I |
| Lydia E. Pinkham Med. Co. | II |
| Erie Medical Co | IV |
| Ruckel & Hendel | VI |
| Ayer's Cherry Pectoral | VI |
| Turf, Field & Farm Ass'n | VIII |
| Herba Vita | IX |
| J. H. Bunnell & Co | X |
| Royal Baking Powder | XI |
| I. S. Johnson & Co. | XI |

